# Polyphenols and Cannabidiol Modulate Transcriptional Regulation of Th1/Th2 Inflammatory Genes Related to Canine Atopic Dermatitis

**DOI:** 10.3389/fvets.2021.606197

**Published:** 2021-03-05

**Authors:** Marcella Massimini, Elena Dalle Vedove, Benedetta Bachetti, Francesco Di Pierro, Cataldo Ribecco, Claudio D'Addario, Mariangela Pucci

**Affiliations:** ^1^R&D Division, C.I.A.M. Srl, Ascoli, Italy; ^2^Velleja Research, Milan, Italy; ^3^Faculty of Bioscience and Technology for Food, Agriculture and Environment, University of Teramo, Teramo, Italy

**Keywords:** epigenetic, polyphenols, phytocannabinoids, skin inflammation, veterinary research

## Abstract

Canine atopic dermatitis (AD) is a multifactorial allergic disease associated with immune and abnormal skin barrier dysfunction and it is one of the primary causes of pruritus. Using a novel *in vitro* model of AD, here we tried to revert the alteration of transcriptional regulation of AD canine key genes testing a nutraceutical mixture containing flavonoids, stilbene, and cannabinoids, which are already well-known for their applications within dermatology diseases. The nutraceutical mixture induced in inflamed cells a significant downregulation (*p* < 0.05) of the gene expression of *ccl2, ccl17*, and *tslp* in keratinocytes and of *ccl2, ccl17*, and *il31ra* in monocytes. Consistent with the observed alterations of *tslp, ccl2, ccl17*, and *il31ra* messenger RNA (mRNA) levels, a significant increase (*p* < 0.05) of DNA methylation at specific CpG sites on the gene regulatory regions was found. These results lay the foundation for the use of these natural bioactives in veterinary medicine and provide a model for deeper understanding of their mechanisms of action, with potential translation to human research.

## Introduction

In veterinary dermatology practice, canine atopic dermatitis (AD) is a multifactorial allergic disease that affects up to 27% of dogs; it is associated with immune and abnormal skin barrier dysfunction and it is one of the primary causes of pruritus (1). Many pharmacological options for its treatment already exist; however, due to elevated costs, side effects, and/or a long lag phase, treatments based on natural compounds are constantly being developed (2). In fact, due to their low toxicity and high efficacy, nutraceuticals are helpful for AD prevention and treatment (3).

Nutrigenomic studies in humans and animal models have clearly shown that nutraceuticals can influence signaling processes, cell apoptosis, metabolism, immune regulation, and modulate gene expression through epigenetic control (4, 5). Taking into account epigenetic modulations within AD and considering the importance of environment in disease progression, food supplements can delay the onset and improve the prognosis of the disease, giving support when anti-inflammatory feedback mechanisms fail to switch off, thereby avoiding an inflammatory over-response (6).

Atopic dermatitis treatment represents a challenge also in human medicine, given the high incidence and the substantial psychosocial burden involved; for these reasons, many studies are focused on characterizing the disease pathomechanisms in our and other species to find an optimal preclinical model for drug development (7). Transcriptomic analysis was performed for dogs in both skin biopsies from spontaneously occurring AD and from lesions induced by epicutaneous allergen challenge in *Dermatophagoides farinae* house dust mite-sensitized subjects (8–10). AD-related hypersensitivity is originally considered to be a Th2-polarized lymphocyte response, especially in the clinical acute phase of the disease; a large number of Th2 genes are upregulated in canine AD models, especially in those induced by house dust mites (11). It is known, to date, that, AD is often associated with multiple lymphocyte phenotypes characterized by the production of a complex variety of cytokines, including both Th1 and Th2 response mediators (7, 10). Increased mRNA levels of interleukin (IL)-4 and other Th2-related interleukins, such as IL-13 and IL-31, were seen in non-lesional and lesional atopic dogs (10), but recent *ex vivo* studies showed an high presence of interferon gamma (IFN-γ), the principal mediator of the Th1 response, in whole blood samples and skin biopsies from atopic dogs (12–14).

On the basis of the abovementioned mechanisms, advanced *in vitro* models were developed to enhance preclinical study for drug discovery, with the aim of decreasing *in vivo* tests (15). Our purpose was to test the effects of a nutraceutical mixture in a canine AD *in vitro* model, which takes into account the Th1/Th2 inflammation. Keratinocytes and monocytes were chosen for their significant involvement in AD progression (16, 17). In particular, the efficacy of the mixture was evaluated in terms of its ability to modulate the transcriptional regulation of the Th1-/Th2-related genes, known to lead to a massive migration and maturation of blood leukocytes at the site of injury and to an increase of itch stimulus in AD (10, 16).

The active principles included in the mixture are two polyphenols, flavone luteolin and stilbene piceatannol, and cannabidiol. They were selected for their already known involvement in reducing skin inflammation and allergic diseases (18, 19).

## Materials and Methods

### Materials

Chemicals were of the purest analytical grade. IFN-γ (781-CG) and IL-13 (5894-CL) were purchased from R&D System (Minneapolis, MN, USA), and IL-4 (754-CL) and IL-31 (59591) from Novus Biologicals (Centennial, CO, USA). Luteolin (sc-203119A) was from Santa Cruz Biotechnology (Dallas, TX, USA), while piceatannol (S3026) and cannabidiol (C6395) were from Selleckchem (Houston, TX, USA) and Sigma-Aldrich (St. Louis, MO, USA), respectively.

### Cell Culture and Inflammatory Stimulation

Canine monocyte-macrophage, DH82 (ATCC, Manassas, VA, USA) and canine epidermal keratinocyte progenitor (CPEK; CELLnTEC, Bern, Switzerland) cell lines were grown at 37°C in humidified 5% CO_2_ atmosphere, in accordance with the manufacturer's recommended protocols. The DH82 cell line was seeded in complete growth medium containing Eagle's minimum essential medium (EMEM), 15% fetal bovine serum, and 1% penicillin/streptomycin; CPEK cells were seeded in complete epidermal keratinocyte medium (CELLnTEC, Bern, Switzerland) supplemented with 1% penicillin/streptomycin. At the optimal culture conditions and considering each cell line doubling time, 5 × 10^5^ DH82 cells and 2.5 × 10^5^ CPEK cells were seeded onto 60-mm Petri dishes; after 24 h, cells were prestimulated with the Th1 cytokine IFN-γ (5 ng/ml) for 48 h, then subjected to the inflammatory stimulation (“inflamed”) with a Th2 interleukin mixture composed of IL-4 (50 ng/ml), IL-13 (50 ng/ml), and IL-31 (50 ng/ml) for 4 h and 8 h. Inflammatory stimuli and dosage have been chosen from the literature with the aim to upregulate canine AD marker genes (16, 17, 20, 21).

### Nutraceutical Dosage Information and Effects on Cell Viability

Inflamed cells were treated with the nutraceutical mixture containing luteolin, piceatannol, and cannabidiol at doses established through the literature and MTT assay results (22–24). The effects of the individual mixture components on the skin inflammation markers were already known in the literature so their synergic ability has been evaluated here (25–27). Piceatannol [dissolved in dimethyl sulfoxide (DMSO)] and luteolin and cannabidiol (dissolved in methanol) were added directly to the culture medium at the following concentrations (Mix 1: cannabidiol 10 μM, luteolin 10 μM, and piceatannol 10 μM; Mix 2: 10:25:25; Mix 3: 10:50:50; and Mix 4: 10:100:100 μM) for 4 h and 8 h. The culture medium containing vehicles alone was added to controls under the same conditions.

To determine the mixtures effect on the cell proliferation, 7.5 × 10^2^ cells/well were seeded onto 96-well plates. At the beginning of the experiment, the complete growth medium was replaced with fresh medium containing the nutraceutical mixture or vehicles. After 4 h and 8 h of incubation, the supernatants were replaced with 0.1 ml of the fresh medium without phenol red, containing 0.5 mg/ml of MTT; the plates were then returned to the incubator for 4 h and were gently shaken occasionally. Crystals of formazan (the MTT metabolic product) were solubilized by 0.1 ml ethanol/DMSO 1:1 lysis buffer and spectrophotometrically quantified at a wavelength of 570 nm with the reference at a wavelength of 695 nm. The differences in the cell growth were measured as a percentage of the growth rates of treated cells compared to untreated cultures.

### Gene Expression

Total RNA was extracted from ≃1 × 10^6^ cells using the All Prep DNA/RNA Mini Kit (QIAGEN, Hilden, Germany) following the manufacturer's recommended protocol. Starting with 1 μg of RNA templates, first strand complementary DNA (cDNA) was synthesized using the RevertAid H Minus First Strand cDNA Synthesis Kit (Thermo Fisher Scientific, Waltham, MA, USA). The relative abundance of the canine AD marker genes was evaluated by real-time PCR using the SensiFAST SYBR Lo-ROX kit (Bioline, London, UK) on a 7500 Fast Real-Time PCR System (Thermo Fisher Scientific, Waltham, MA, USA) in both cell lines. The primers used for the amplification are reported in Table 1, and all of the data were normalized to the endogenous reference gene β-actin.

**Table 1:** Primer sequences used to analyze mRNA expression levels of canine genes.

**Gene**	**Forward (5′ → 3′)**	**Reverse (5′ → 3′)**
*il4r*	GAAAGGATGGTGGGATCAGA	CAAGCTCCTGCCCTGTCTAC
*il31ra*	ATGGATGCTCCTTCTACTCTGTAAACT	CAGGAAATGTTCTCAGGCTTAGC
*il31*	CTCTCCCACACAGGACCATC	TGGGAGGACAGCAAGGTTTC
*ccl2*	CCTGCTGCTATACACTCA	GCTTCTTTGGGACACTTG
*ccl5*	CAGTCGTCTTTGTCACCCGA	TGTACTCCCGCACCCATTTC
*ccl17*	GCCATCGTGTTTGTAACT	CTCCCTTCCAGGTTCTTTGT
*tslp*	AGTACACGGGGTGGCTGA	GTCATTTACCAAGCCCTGGA
*ctss*	AAAGCGAGCTGCCACATGT	TTAAGGCATCTTCACTGCCAAA
β-*actin*	TTCCGCTGCCCAGAGGCTCT	GCTCAGGGGGTGCGATGATCTTG

### DNA Methylation Analysis by Pyrosequencing

The DNA methylation levels of the specific CpG sites were determined using bisulfite-converted DNA from DH82 and CPEK cells by pyrosequencing. The genomic DNA was simultaneously purified with RNA as mentioned above, and DNA (1 μg) was subjected to bisulfite modification using the EZ DNA Methylation Kit (Zymo Research, Irvine, CA, USA).

**Figure 1:**
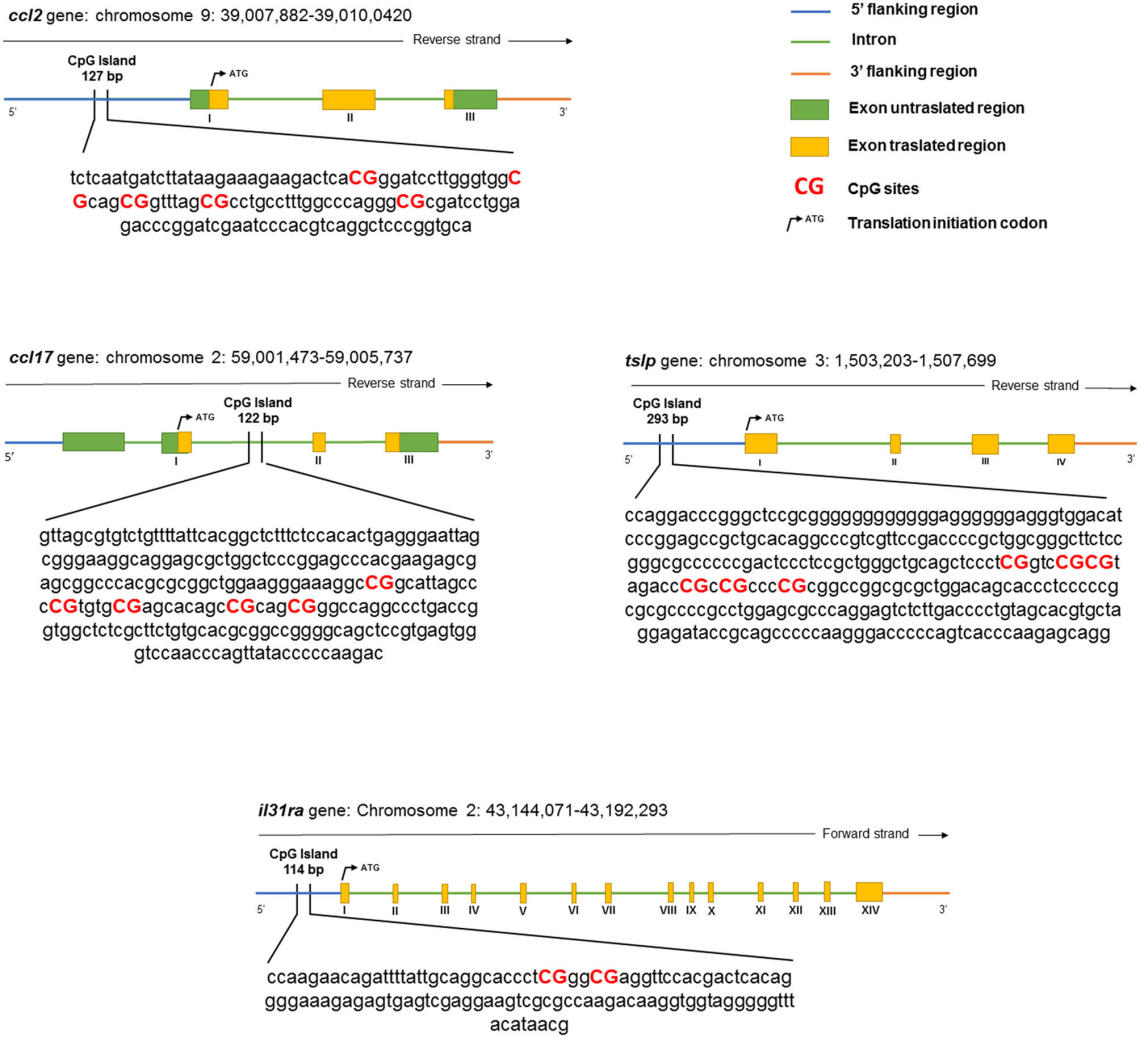
The schematic representation of the analyzed canine genes.

The bisulfite-treated DNA was amplified using the PyroMark PCR Kit (QIAGEN, Hilden, Germany) according to the protocol of the manufacturer. The PCR conditions were as follows: 95°C for 15 min, followed by 45 cycles of 94°C for 30 s, 56°C for 30 s, 72°C for 30 s, and finally, 72°C for 10 min. After product quality verification by 1.8% agarose electrophoresis, the pyrosequencing methylation analysis was conducted using the PyroMark Q24 (QIAGEN, Hilden, Germany). The level of methylation was analyzed using PyroMark Q24 software (QIAGEN, Hilden, Germany), which calculates the methylation percentage using the following formula: methylated cytosine/methylated cytosine + unmethylated cytosine. Quantitative methylation results were considered, both as a percentage of single CpG sites and as an average of the methylation percentage of all the investigated CpG sites. A schematic representation of a CpG island and the details of the pyrosequencing assay are given in Figure 1 and Table 2, respectively.

**Table 2:** Primer sequences used to analyze DNA methylation levels of canine genes.

**Gene**	**Forward (5**′** → 3**′**)**
*ccl2*	GGGAAGTTTTGTGTATTTTATAGGTATTG
*ccl17*	AGGGAATTAGAGGGAAGGTAGGA
*il31ra*	GAGGGTTTAAGAATAGATTTTATTGTAGGT
*tslp*	TTTTAGTAAGTGTTATAGGGGTTAAGAGAT
**Gene**	**Reverse biotinylated (5′** **→** **3′)**
*ccl2*	AACAAAAAAAAAAACAAACTCCATAC
*ccl17*	TAAAAACCCCCAACAACCTCC
*Il31ra*	AAACCCCCTACCACCTTATCTT
*tslp*	ACTCCCTCCCCTAAACTACAAC
**Gene**	**Sequencing (5′** **→** **3′)**
*ccl2*	AATGATTTTATAAGAAAGAAGATT
*ccl17*	GGTTGGAAGGGAAAG
*il31ra*	AATAGATTTTATTGTAGGTATT
*tslp*	GGGGTTAAGAGATTTTTG

### Statistical Analysis

Data were expressed as the mean ± SEM for each group and were analyzed using the GraphPad Prism version 8.0.0 (GraphPad Software, San Diego, CA, USA). Data from the MTT assay were statistically evaluated by the one-way ANOVA followed by the Bonferroni's multiple comparison test for further examination of group differences (*p* < 0.05).

The gene expression was analyzed by three-way ANOVA using the factors “inflammation,” “nutraceuticals treatment,” and “time” followed by the Bonferroni's *post-hoc* test to follow up on significant interactions or main effects (*p* < 0.05) from the factorial ANOVA. The effects of the nutraceutical treatment on inflamed cells were evaluated using the two-way ANOVA, and the one-way ANOVA was used in cases of no interaction between factors (nutraceuticals and time). The statistical differences with respect to average DNA methylation changes at gene promoters were analyzed by multiple *t*-tests (*p* < 0.05).

## Results

### Effects of Inflammation on AD Markers Gene Expression

The effects evoked by the inflammatory stimulation on canine DH82 and CPEK cells on the expression of selected genes were assessed by real-time PCR, and the results are shown in Table 3 (CPEK cell line; “keratinocytes”) and **4** (DH82 cell line; “monocytes”). Selected genes derived from a previous screening aimed to sort markers of inflammation in stimulated cells (the data regarding model time course are not shown). Three-way ANOVA showed, among the selected genes, an increase in mRNA levels in inflamed cells (Inflamed: yes; Mix: no) with respect to the control (Inflamed: no; Mix: no). The Bonferroni's *post-hoc* test results are shown in Tables 3, 4.

**Table 3:** The effects of the nutraceutical treatment on the relative expression (fold change) of canine AD marker genes on CPEK keratinocytes.

**CPEK**								
**Time**	**4 h**				**8 h**			
**Inflamed**	**No**		**Yes**		**No**		**Yes**	
**Mix**	**No**	**Yes**	**No**	**Yes**	**No**	**Yes**	**No**	**Yes**
*ccl2*	1.05 ± 0.16	1.39 ± 0.39	**9.65** ± **0.34^a^**	**5.74** ± **0.77^a^^,^^b^^,^^c^**	0.62 ± 0.04	0.74 ± 0.31	**16.53** ± **1.44^a^^,^^b^^,^^c^^,^^e^**	**3.67** ± **0.88^a^^,^^d^^,^^e^^,^^f^**
*ccl5*	1.00 ± 0.19	1.10 ± 0.07	**3.47** ± **0.88^a^**	**4.20** ± **1.42^a^^,^^b^**	0.62 ± 0	1.21 ± 0	**4.82** ± **0.89^a^^,^^b^^,^^e^**	**2.68** ± **0.4^f^**
*ccl17*	1.03 ± 0.16	0.50 ± 0.12	**1.77** ± **0.2^a^**	**0.56** ± **0.03^c^**	1.10 ± 0.24	0.51 ± 0.01	**1.88** ± **0.32^a^^,^^b^^,^^e^**	**0.78** ± **0.4^f^**
*tslp*	1.02 ± 0.41	0.30 ± 0.11	**2.17** ± **0.81^a^**	**0.48** ± **0.23^c^**	0.40 ± 0.07	0.27 ± 0.05	**1.41** ± **0.04^b^**	0.50 ± 0.24
*il31r*	1.02 ± 0.22	1.15 ± 0.46	1.73 ± 0.1	1.52 ± 0.56	1.00 ± 0.02	1.05 ± 0.25	**4.74** ± **0.46^a^^,^^b^^,^^c^^,^^e^**	**4.72** ± **0.91^a^^,^^d^^,^^e^**
*il4r*	1.00 ± 0.07	1.03 ± 0.11	**2.36** ± **0.14^a^**	**2.25** ± **0.21^a^^,^^b^**	0.29 ± 0.02	0.37 ± 0.1	**0.93** ± **0.22^c^**	**0.95** ± **0.41^d^**
*ctss*	1.04 ± 0.3	1.03 ± 0.2	2.11 ± 0.29	2.00 ± 0.48	0.74 ± 0.03	0.88 ± 0.35	**2.29** ± **0.42^b^^,^^e^**	1.87 ± 0.86
*il31*	1.07 ± 0.27	0.62 ± 0.19	0.91 ± 0.1	0.72 ± 0.04	0.70 ± 0.03	0.47 ± 0.03	1.11 ± 0.43	1.13 ± 0.15

a p < 0.05 vs. no inflamed 4 h no mix 4 h;

b p < 0.05 vs. no inflamed 4 h yes mix 4 h;

c p < 0.05 vs. yes inflamed 4 h no mix 4 h;

d p < 0.05 vs. yes inflamed 4 h yes mix 4 h;

e p < 0.05 vs. no inflamed 8 h no mix 8 h;

f *p < 0.05 vs. yes inflamed 8 h no mix 8 h*.

**Table 4:** The effects of the nutraceutical treatment on the relative expression (fold change) of canine AD marker genes on DH82 monocytes.

**DH82**								
**Time**	**4 h**				**8 h**			
**Inflamed**	**No**		**Yes**		**No**		**Yes**	
**Mix**	**No**	**Yes**	**No**	**Yes**	**No**	**Yes**	**No**	**Yes**
*ccl2*	1.01 ± 0.21	0.37 ± 0.06	**3.52** ± **0.61^a^**	**1.92** ± **0.11^b^^,^^c^**	1.17 ± 0.16	0.27 ± 0.03	**2.60** ± **0.71^a^^,^^b^^,^^e^**	1.80 ± 0.12
*ccl5*	0.98 ± 0.14	1.63 ± 0.24	**35.89** ± **4.94^a^**	**36.31** ± **1.69^a^^,^^b^**	0.99 ± 0.2	1.75 ± 0.19	**27.91** ± **1.45^a^^,^^b^^,^^c^^,^^e^**	**30.34** ± **1.03^a^^,^^d^^,^^e^**
*ccl17*	0.95 ± 0.24	0.72 ± 0.09	**17.06** ± **3.06^a^**	3.27 ± 0.12	1.40 ± 0.22	0.79 ± 0.14	**39.90** ± **4.48^a^^,^^b^^,^^c^^,^^e^**	**3.98** ± **0.83^f^**
*tslp*	1.03 ± 0.25	1.14 ± 0.23	**3.30** ± **0.64^a^**	**3.30** ± **0.79^a^^,^^b^**	0.94 ± 0.28	1.01 ± 0.12	**1.69** ± **0.09^c^**	**1.65** ± **0.11^d^**
*il31r*	0.97 ± 0.32	1.69 ± 0.24	**10.45** ± **0.52^a^**	**4.90** ± **1.72^a^^,^^b^^,^^c^**	1.04 ± 0.13	0.44 ± 0.1	**4.41** ± **1.14^a^^,^^b^^,^^c^^,^^e^**	**3.45** ± **0.56^a^^,^^e^**
*ctss*	1.02 ± 0.2	1.20 ± 0.34	**3.58** ± **0.53^a^**	**3.42** ± **0.43^a^^,^^b^**	0.98 ± 0.14	1.29 ± 0.54	**3.21** ± **1.02^a^^,^^b^^,^^e^**	**3.38** ± **0.84^a^^,^^e^**
*il4r*	1.03 ± 0.3	1.20 ± 0.72	1.66 ± 0.53	1.43 ± 0.19	1.06 ± 0.17	1.13 ± 0.59	0.90 ± 0.19	1.02 ± 0.17
*il31*	1.03 ± 0.11	0.87 ± 0.35	1.22 ± 0.1	1.15 ± 0.05	1.19 ± 0.16	1.36 ± 0.18	**1.59** ± **0.05^a^^,^^b^**	1.36 ± 0.07

a p < 0.05 vs. no inflamed 4 h no mix 4 h;

b p < 0.05 vs. no inflamed 4 h yes mix 4 h;

c p < 0.05 vs. yes inflamed 4 h no mix 4 h;

d p < 0.05 vs. yes inflamed 4 h yes mix 4 h;

e p < 0.05 vs. no inflamed 8 h no mix 8 h;

f *p < 0.05 vs. yes inflamed 8 h no mix 8 h*.

### Effects of the Nutraceutical Mixture on Cell Viability

**Figure 2:**
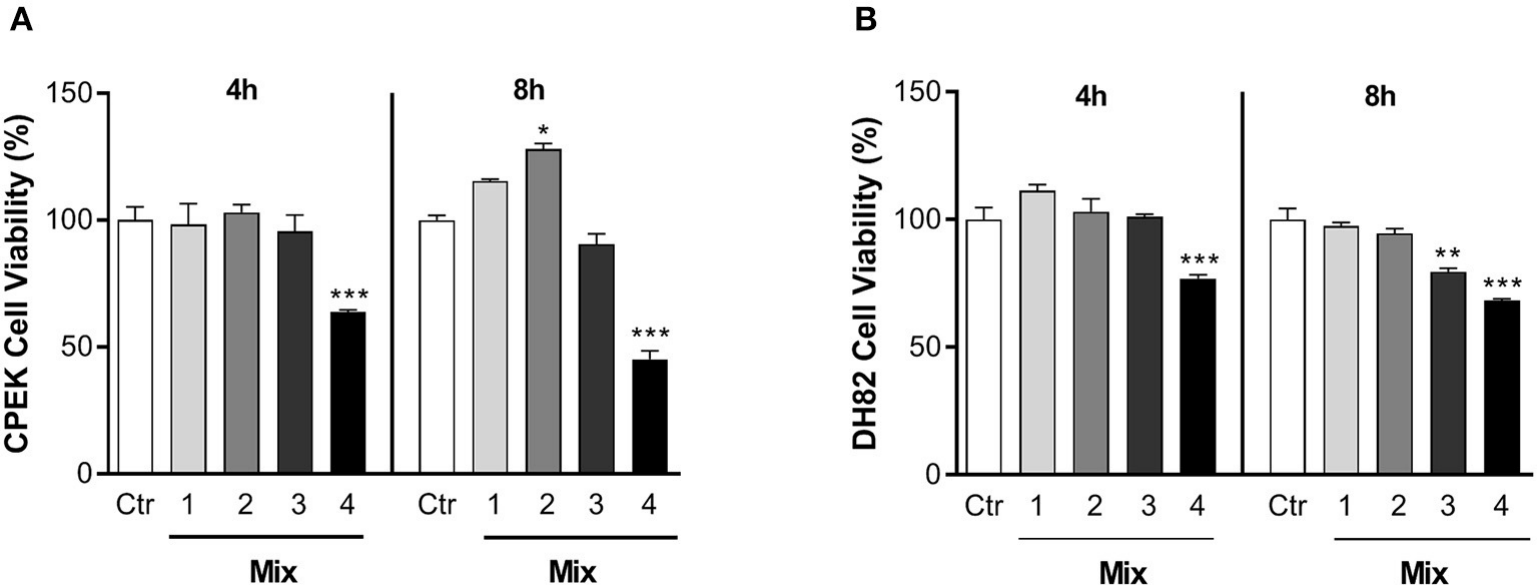
The effects of nutraceutical mixtures on the viability of inflamed cells. Inflamed **(A)** CPEK and **(B)** DH82 cells were treated with nutraceutical mixtures containing an increasing ratio of luteolin and piceatannol to cannabidiol for 4 and 8 h. Cell viability is expressed as the percentage of untreated cells (vehicle alone = 100% cell viability). The bar graphs show the mean + SEM of three independent experiments performed in triplicate. The statistically significant differences between experimental conditions and untreated control cells are shown by asterisks (*p < 0.01, **p < 0.01, ***p < 0.001).

The effects of different nutraceutical mixtures on cell viability were tested on CPEK and DH82 cells (Figure 2). Inflamed cells, previously stimulated as shown in section Materials and Methods, were exposed to different concentrations of cannabidiol, luteolin, and piceatannol (Mix 1: cannabidiol 10 μM, luteolin 10 μM, and piceatannol 10 μM; Mix 2: 10:25:25 μM; Mix 3: 10:50:50 μM, and Mix 4: 10:100:100 μM) for 4 h and 8 h. One-way ANOVA showed that the exposure to the nutraceutical mixtures induced a significant change in the percentage viability of CPEK cells [*F*_(9, 20)_ = 30.45], and *post-hoc* comparisons using the Bonferroni's test revealed a significant decrease in the groups treated for 4 and 8 h with Mix 4 with respect to control (4 h = 63.80 ± 1.17, *p* < 0.001; 8 h = 45.04 ± 5.81, *p* < 0.001). Moreover, 8 h of exposure to Mix 2 induced a significant increase in cell viability (127.96 ± 3.95; *p* = 0.0081) (Figure 2A).

The nutraceutical mixtures induced changes in DH82 cell viability [*F*_(9, 20)_ = 22.58; *p* < 0.001]. The Bonferroni's comparison test revealed a decrease in cell viability with respect to control after Mix 4 treatment for 4 and 8 h (4 h = 76.87 ± 2.45, *p* < 0.001; 8 h = 68.22 ± 1.16, *p* < 0.001). Moreover, a significant decrease in the percentage viability was observed in the DH82 cells treated with Mix 3 for 8 h (79.59 ± 2.37; *p* = 0.0029) (Figure 2B). Due to its ability to enhance CPEK viability without provoking cell death in the DH82 cell line, Mix 2 was chosen for all the subsequent experiments.

### Effects of the Nutraceutical Mixture on Transcriptional Regulation in Inflamed Cells

**Figure 3:**
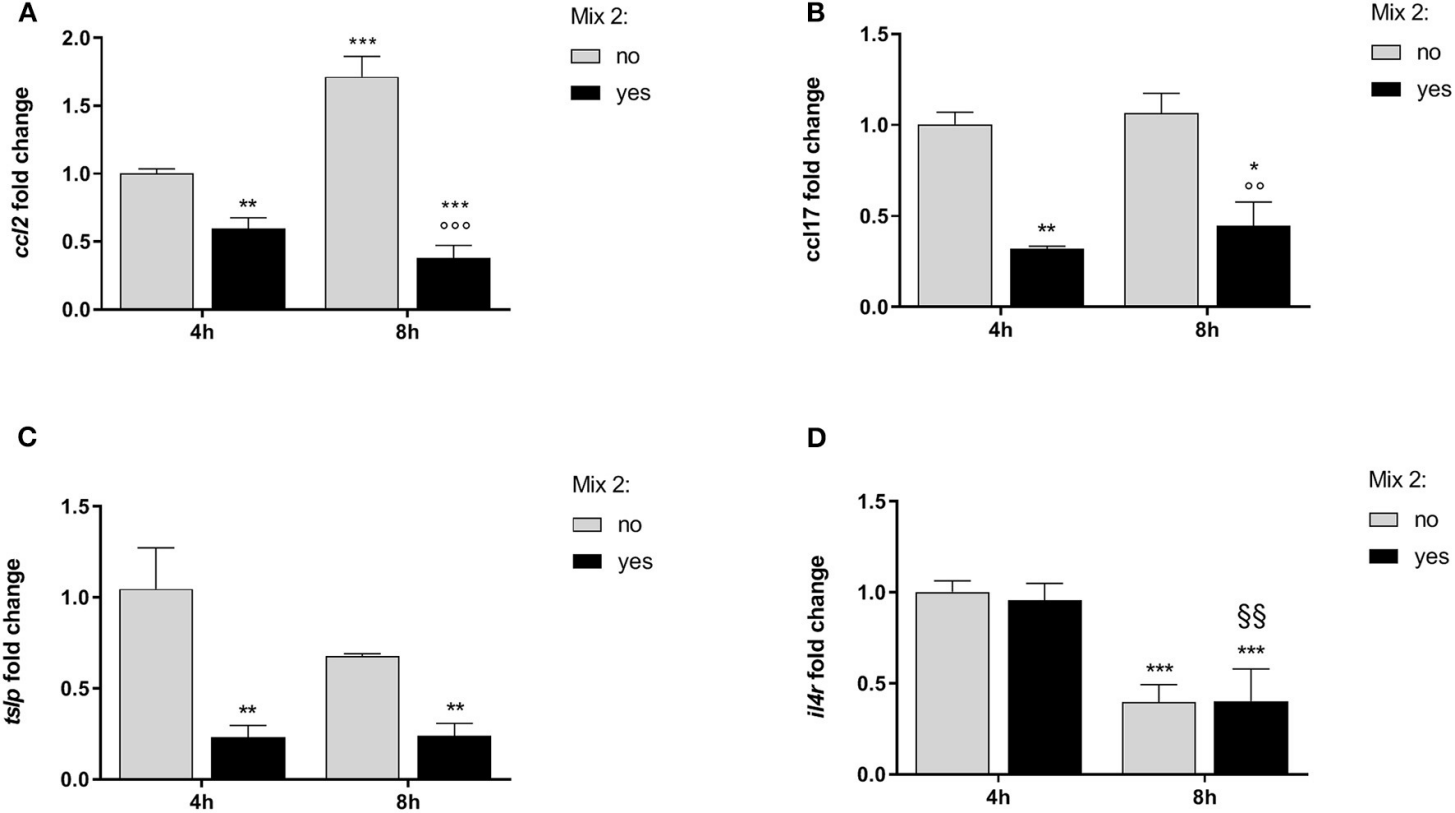
The relative expression of canine atopic dermatitis (AD) marker genes after the nutraceutical mixture treatment in CPEK cells. Inflamed cells were treated (Mix yes) or not (Mix no) with the nutraceutical mixture (Mix 2) for 4 and 8 h. The bar graphs show the mean + SEM of three independent experiments performed in triplicate. The gene expression of *ccl2* **(A)**, *ccl17* **(B)**, *tslp* **(C)**, and *il4r* **(D)** was calculated using the delta–delta Ct (ΔΔCt) method. The statistically significant differences between experimental conditions are reported as follows: *p < 0.05, **p < 0.01, ***p < 0.001 vs. no mix 4 h; §§ *p* < 0.01, vs. yes mix 4 h; °°°*p* < 0.001 vs. no mix 8 h.

**Figure 4:**
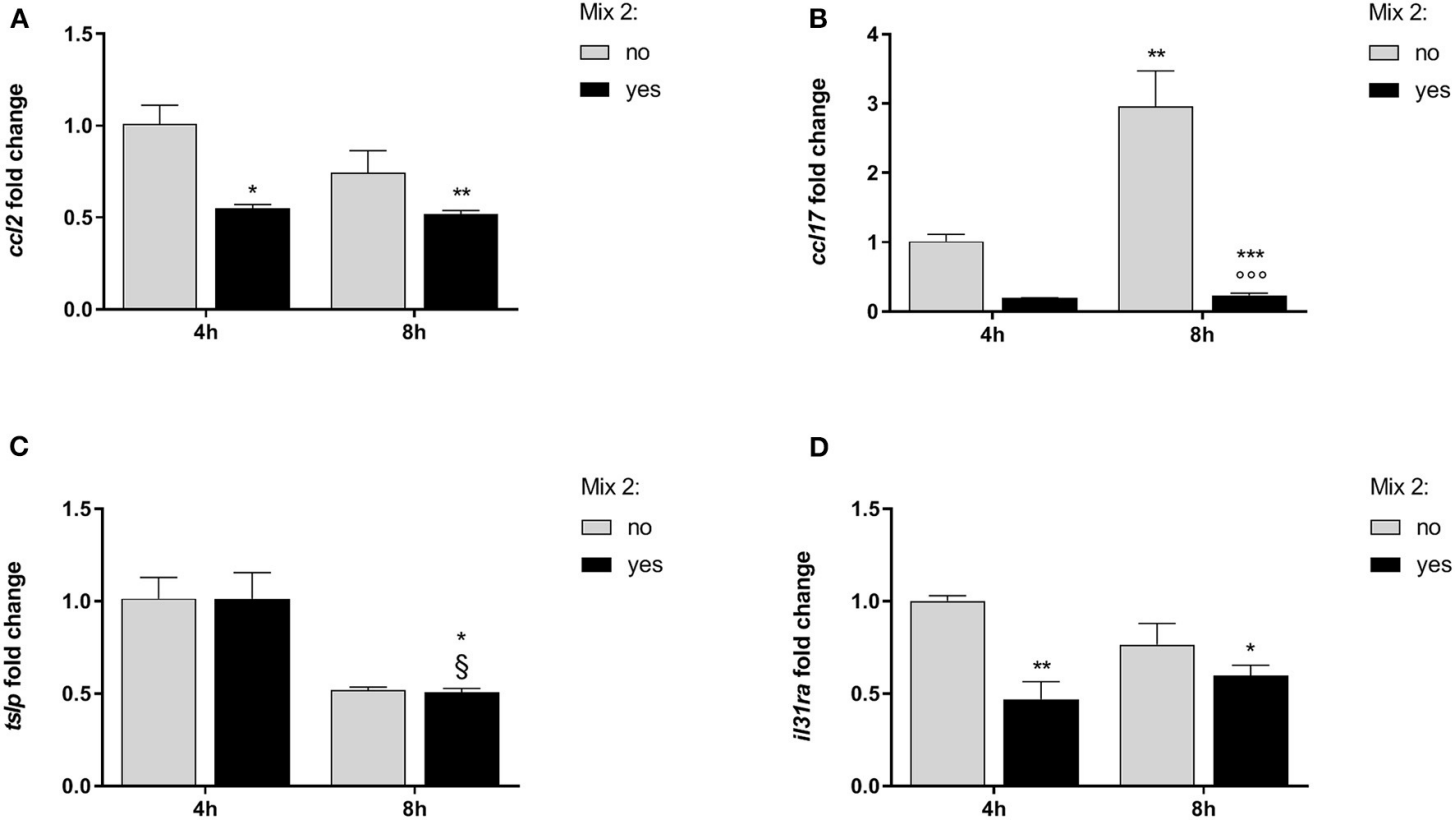
The relative expression of canine AD marker genes after the nutraceutical mixture treatment in DH82 cells. Inflamed cells were treated (Mix yes) or not (Mix no) with the nutraceutical mixture (Mix 2) for 4 and 8 h. The bar graphs show the mean + SEM of three independent experiments performed in triplicate. The gene expression of *ccl2* **(A)**, *ccl17* **(B)**, *tslp* **(C)**, and *il31ra* **(D)** was calculated using the delta–delta Ct (ΔΔCt) method. The statistically significant differences between experimental conditions are reported as follows: *p < 0.05, **p < 0.01, ***p < 0.001 vs. no mix 4 h; ^§^*p* < 0.05 vs. yes mix 4 h.

The consequences of the nutraceutical mixture (Mix 2) treatment after 4 and 8 h on the inflamed cells are shown in Figure 3 and Table 3 (CPEK) and Figure 4 and Table 4 (DH82).

The statistical analysis using two-way ANOVA showed that *ccl2* mRNA levels in inflamed CPEK cells were affected by time [*F*_(1, 8)_ = 19.32; *p* = 0.0023] and Mix 2 treatment [*F*_(1, 8)_ = 234.0; *p* < 0.001], with a significant interaction between these two factors [*F*_(1, 8)_ = 66.62; *p* < 0.001]. The Bonferroni's *post-hoc* test results are shown in Figure 3A.

An alteration of *ccl17* expression was observed in CPEK cells inflamed and exposed to the Mix 2 for 4 and 8 h (Figure 3B). The two-way ANOVA showed that mRNA levels were affected by the Mix 2 treatment [*F*_(1, 8)_ = 50.79; *p* < 0.001] but not by time [*F*_(1, 8)_ = 1.03; *p* = 0.3403], without an interaction between the two factors [*F*_(1, 8)_ = 0.11; *p* = 0.7502]. The one-way ANOVA further showed that the *ccl17* mRNA levels in CPEK cells were significantly affected by treatment [*F*_(3, 8)_ = 17.31; *p* < 0.001], and *post-hoc* comparisons are indicated in Figure 3B.

The statistical analysis using the two-way ANOVA showed that *tslp* expression in CPEK cells was also affected by treatment [*F*_(1, 8)_ = 26.19; *p* < 0.001] but not by time [*F*_(1, 8)_ = 2.16; *p* = 0.18], without a significant interaction between these two factors [*F*_(1, 8)_ = 2.37; *p* = 0.16]. The one-way ANOVA further showed that the *tslp* gene expression was affected by treatment [*F*_(3, 8)_ = 10.24; *p* = 0.004] (Figure 3C). The Bonferroni's *post-hoc* test results are shown in Figure 3C.

The two-way ANOVA showed that the *il4r* mRNA levels in CPEK cells were not affected by the Mix 2 treatment [*F*_(1, 8)_ = 0.08; *p* = 0.80] but were affected by time [*F*_(1, 8)_ = 76.64; *p* < 0.001], without an interaction between the two factors [*F*_(1, 8)_ = 0.15; *p* = 0.71]. As mentioned earlier, time affected the *il4r* gene expression [*F*_(3, 8)_ = 25.62; *p* < 0.001], and *post-hoc* comparisons are indicated in Figure 3D.

In inflamed monocytes, the *ccl2* gene expression was affected by the Mix 2 treatment [*F*_(1, 8)_ = 18.80; *p* = 0.002] but not by time [*F*_(1, 8)_ = 3.51; *p* = 0.10], without an interaction between the factors [*F*_(1, 8)_ = 2.12; *p* = 0.18]. The one-way ANOVA further showed that the *ccl2* mRNA levels were affected by treatment [*F*_(3, 8)_ = 8.146; *p* = 0.0082]. The Bonferroni's *post-hoc* test results are shown in Figure 4A.

The gene expression of *ccl17* in DH82 cells was affected by time [*F*_(1, 8)_ = 14.04; *p* = 0.006] and Mix 2 treatment [*F*_(1, 8)_ = 44.48; *p* < 0.001] with a positive interaction between factors [*F*_(1, 8)_ = 12.87; *p* = 0.007]. The *post-hoc* comparison test results are reported in Figure 4B.

The expression of *tslp* in DH82 cells was dependent on time [*F*_(1, 8)_ = 29.59; *p* < 0.001] but was not affected by the Mix 2 treatment [*F*_(1, 8)_ = 0.004; *p* = 0.95], with no interaction between factors [*F*_(1, 8)_ = 0.004; *p* = 0.95]. The one-way ANOVA further showed that the *tslp* gene expression was affected by time [*F*_(3, 8)_ = 9.86; *p* = 0.005] (Figure 4C). The Bonferroni's *post-hoc* test results are shown in Figure 4C.

Finally, the *il31ra* expression in DH82 cells was affected by treatment [*F*_(1, 8)_ = 18.68; *p* = 0.002] but not by time [*F*_(1, 8)_ = 0.4520; *p* = 0.5203], with no interaction between factors [*F*_(1, 8)_ = 5.07; *p* = 0.05]. The one-way ANOVA further showed that the *il31ra* gene expression was affected by the treatment [*F*_(3, 8)_ = 8.07; *p* = 0.008], and the *post-hoc* comparisons are indicated in Figure 4D.

### Nutraceutical Effects on DNA Methylation

In order to evaluate whether the epigenetic mechanisms could account for the gene expression changes, we analyzed the DNA methylation at specific promoter regions. The DNA methylation analysis for each CpG site as well as the average values for inflamed cells is shown in Tables 5, 6.

**Table 5:** The DNA methylation (%) changes at inflamed modulated gene promoters after the mixture treatment of inflamed CPEK cells.

**CPEK**					
**Time**		**4 h**		**8 h**	
**Inflamed**		**Yes**			
**Mix**		**No**	**Yes**	**No**	**Yes**
*ccl2*	Site 1	8.33 ± 0.44	8.18 ± 0.24	5.15 ± 4.58	19.23 ± 9.23
	Site 2	81.20 ± 2.00	82.68 ± 0.88	81.53 ± 0.60	59.00 ± 37.03
	Site 3	73.81 ± 1.27	73.84 ± 1.08	65.18 ± 13.94	67.03 ± 17.83
	Site 4	90.98 ± 0.83	91.76 ± 0.48	83.20 ± 15.53	81.54 ± 20.69
	Site 5	91.23 ± 0.60	91.83 ± 1.70	86.71 ± 6.95	79.70 ± 16.44
	Average	69.11 ± 0.65	69.66 ± 0.75	64.35 ± 11.55	61.30 ± 17.46
*ccl17*	Site 1	97.69 ± 2.33	99.33 ± 1.16	98.82 ± 1.11	100.00 ± 0.00
	Site 2	88.55 ± 3.47	90.36 ± 2.33	92.02 ± 7.03	95.82 ± 3.66
	Site 3	81.44 ± 1.87	**75.22** ± **2.35**^**a**^	78.09 ± 1.63	78.53 ± 7.99
	Site 4	98.47 ± 1.80	100.00 ± 0.00	98.31 ± 2.92	92.56 ± 12.89
	Site 5	88.78 ± 2.76	84.80 ± 7.61	88.41 ± 8.85	84.95 ± 4.45
	Average	90.99 ± 0.93	89.94 ± 2.01	91.15 ± 4.05	90.37 ± 0.91
*tslp*	Site 1	95.06 ± 0.43	**96.43** ± **0.44**^**a**^	95.83 ± 1.79	96.85 ± 1.59
	Site 2	93.97 ± 0.54	94.82 ± 0.58	96,05 ± 2.42	96.05 ± 2.42
	Site 3	80.29 ± 0.94	81.21 ± 2.30	83.17 ± 2.78	83.17 ± 2.78
	Site 4	85.38 ± 1.02	83.79 ± 4.17	87.72 ± 3.80	87.72 ± 3.80
	Site 5	77.05 ± 1.10	71.25 ± 11.77	83.38 ± 9.04	83.38 ± 9.04
	Site 6	92.01 ± 0.89	93.44 ± 0.35	93.66 ± 1.58	93.66 ± 1.58
	Average	87.29 ± 0.31	86.82 ± 1.91	89.96 ± 1.83	90.14 ± 3.14

a *p < 0.05 vs. yes inflamed 4 h no mix 4 h*.

**Table 6:** The DNA methylation (%) changes at inflamed modulated gene promoters after the mixture treatment of inflamed DH82 cells.

**DH82**					
**Time**		**4 h**		**8 h**	
**Inflamed**		**Yes**			
**Mix**		**No**	**Yes**	**No**	**Yes**
ccl2	Site 1	81.20 ± 6.39	87.95 ± 1.80	77.91 ± 5.97	77.43 ± 1.06^b^
	Site 2	79.52 ± 6.16	**91.46** ± **1.19**^**a**^	87.31 ± 4.53	85.29 ± 5.13
	Site 3	90.20 ± 4.96	94.93 ± 0.88	90.41 ± 5.32	93.13 ± 3.28
	Site 4	92.49 ± 3.56	97.06 ± 0.85	96.06 ± 0.38	96.34 ± 0.39
	Site 5	93.51 ± 6.96	95.62 ± 1.52	92.15 ± 4.97	94.60 ± 1.71
	Site 6	76.32 ± 2.95	76.90 ± 3.31	73.88 ± 4.11	**84.39** ± **3.86^b^^,^^c^**
	Average	85.54 ± 3.69	90.65 ± 0.79	86.29 ± 3.30	88.53 ± 0.97
*ccl17*	Site 1	100.00 ± 0.00	99.38 ± 0.88	99.25 ± 1.30	100.00 ± 0.00
	Site 2	93.46 ± 11.32	82.98 ± 15.70	81.25 ± 8.22	74.22 ± 2.79
	Site 3	84.20 ± 2.40	81.70 ± 5.50	**76.27** ± **1.33**^**a**^	**84.17** ± **0.97**^**c**^
	Site 4	99.95 ± 0.09	98.76 ± 2.15	100.00 ± 0.00	98.29 ± 2.20
	Site 5	89.11 ± 1.75	89.12 ± 2.19	86.79 ± 1.81	89.60 ± 1.54
	Average	93.34 ± 2.94	90.39 ± 5.18	88.71 ± 2.05	89.25 ± 2, 38
*il31ra*	Site 1	44.88 ± 1.34	49.25 ± 4.57	50.30 ± 3.27	48.46 ± 0.59
	Site 2	43.07 ± 1.29	**47.21** ± **2.20**^**a**^	47.46 ± 3.59	45.49 ± 1.92
	Average	43.98 ± 0.86	48.23 ± 3.38	48.88 ± 3.38	46.98 ± 1.10

a p < 0.05 vs. yes inflamed 4 h no mix 4 h;

b p < 0.05 vs. yes inflamed 4 h yes mix 4 h;

c *p < 0.05 vs. yes inflamed 8 h no mix 8 h*.

**Figure 5:**
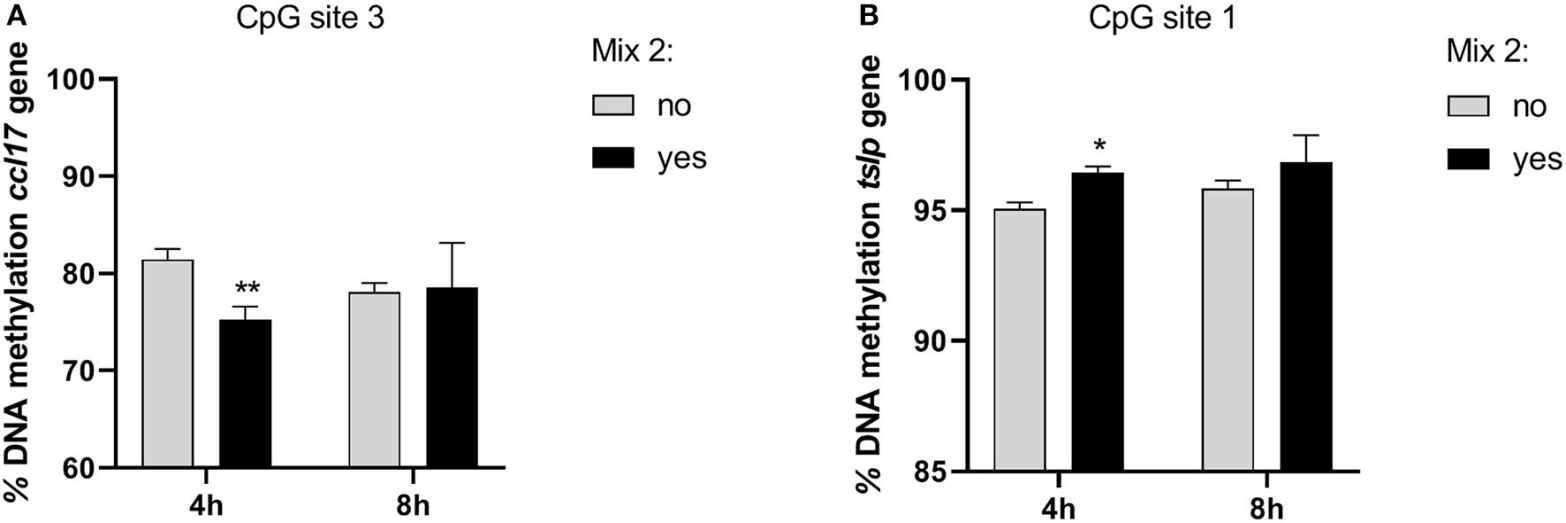
The effects of the nutraceutical mixture on CpG island methylation in CPEK cells. Inflamed cells were treated (Mix yes) or not (Mix no) with the nutraceutical mixture (Mix 2) for 4 and 8 h. The bar graphs represent the mean + SEM of three independent experiments performed in triplicate. The figure shows the methylation percentage with respect to *ccl17* **(A)** and *tslp* **(B)** expressed as methylated cytosine/methylated cytosine + unmethylated cytosine. The statistically significant differences between experimental conditions are reported as follows: *p < 0.05, **p < 0.01.

In the inflamed CPEK cells exposed to the Mix 2 for 4 and 8 h, we failed to observe any overall alteration in the percentage of DNA methylation at *ccl2, ccl17*, and *tslp* gene promoters with respect to the CpG sites analyzed. Multiple *t*-test comparisons showed, in inflamed cells exposed for 4 h to Mix 2, a significant decrease in the percentage of DNA methylation at the third CpG site in the promoter region of *ccl17* (75.22 ± 2.35 Yes Mix vs. 81.44 ± 1.87 No Mix; *p* = 0.02) (Figure 5A) and an increase in the first CpG site of *tslp* evaluated (96.43 ± 0.44 Yes Mix vs. 95.06 ± 0.43 No Mix; *p* = 0.02) (Figure 5B).

**Figure 6:**
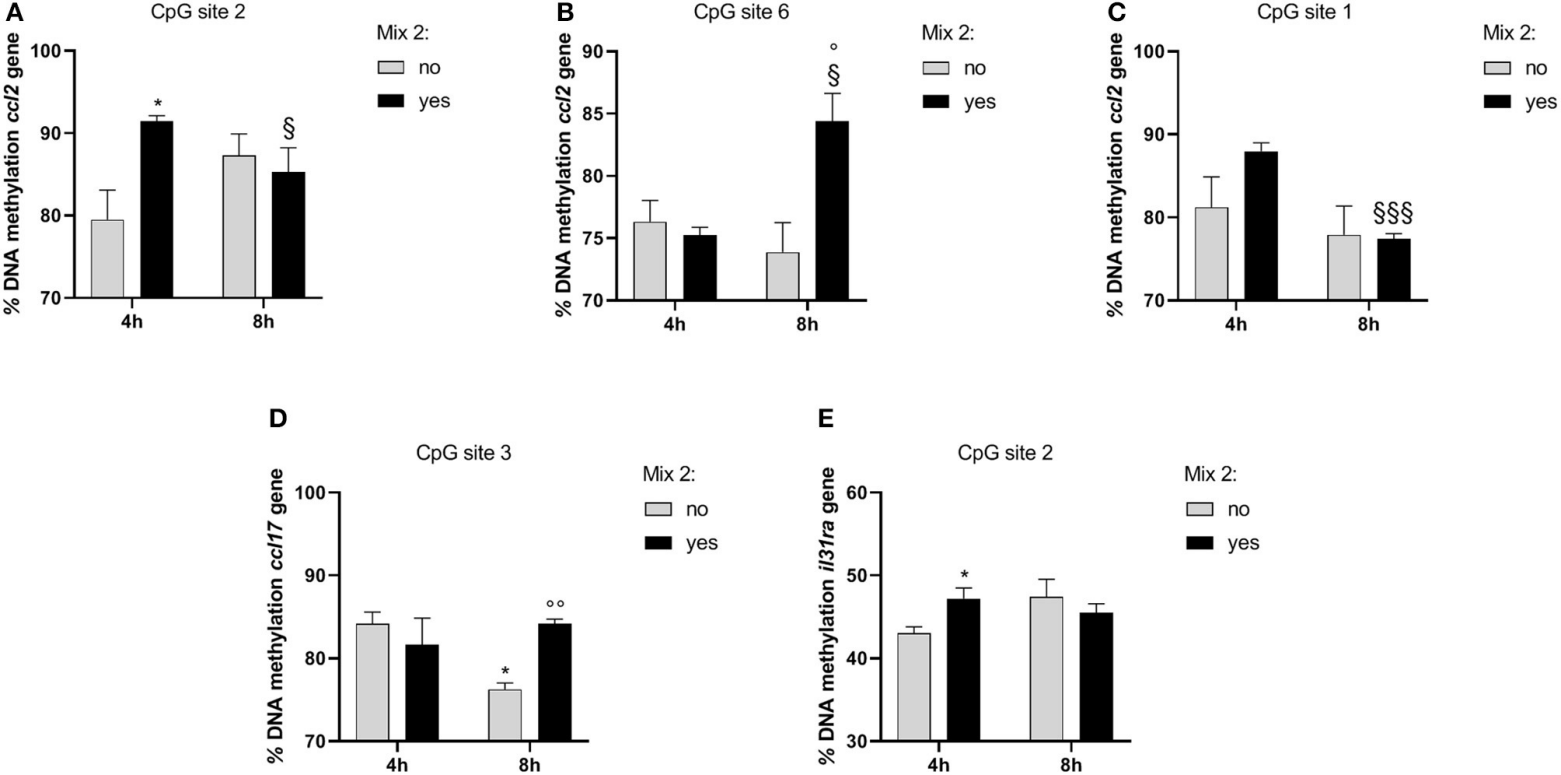
The effects of the nutraceutical mixture on modulated gene CpG island methylation in DH82 cells. Inflamed cells were treated (Mix yes) or not (Mix no) with nutraceutical mixture (Mix 2) for 4 and 8 h. The bar graphs represent the mean + SEM of three independent experiments performed in triplicate. The figure shows the methylation percentage with respect to *ccl2* **(A–C)**, *ccl17* **(D)**, and *il31ra* **(E)** expressed as methylated cytosine/methylated cytosine + unmethylated cytosine. The statistically significant differences between experimental conditions are reported as follows: *p < 0.05; ^§^*p* < 0.05, ^§§§^*p* < 0.001 vs. yes mix 4 h; °*p* < 0.05, °°*p* < 0.01 vs. no mix 8 h.

In the inflamed DH82 cells, the *ccl2* gene promoter showed a substantial methylation increase in CpG site 2 for cells treated for 4 h with respect to untreated cells (Yes Mix 91.46 ± 1.19, No Mix 79.52 ± 6.16; *p* = 0.03) (Figure 6A). Moreover, an increase in the percentage of DNA methylation was observed in CpG site 6 in cells treated for 8 h (Yes Mix 84.39 ± 3.86) with respect to untreated cells (No Mix 73.88 ± 4.11; *p* = 0.03) and 4 h treated cells (76.90 ± 3.31; *p* = 0.016) (Figure 6B). A decrease in the percentage of methylation, after 8 h of Mix 2 exposure, was also observed in site 1 with respect to 4 h Mix 2-treated cells (77.43 ± 1.06 Yes Mix 8 h vs. 87.95 ± 1.80 Yes Mix 4 h; *p* < 0.001) (Figure 6C). The nutraceutical mixture exposure induced a significant increase in the percentage of methylation in *ccl17* site 3 after 8 h (Yes Mix 84.17 ± 0.97 vs. No Mix 76.27 ± 1.33; *p* = 0.001) (Figure 6D). A significant increase in the percentage of methylation in *il31ra* site 2 was observed after 4 h in the treated cells (47.21 ± 2.20 vs. 43.07 ± 1.29 No Mix; *p* = 0.047) (Figure 6E).

Moreover, the significant differences between groups, considering also those not inflamed, were evaluated through the three-way ANOVA, and multiple *t*-test comparisons are shown in Supplementary Tables 1, 2.

## Discussion

**Figure 7:**
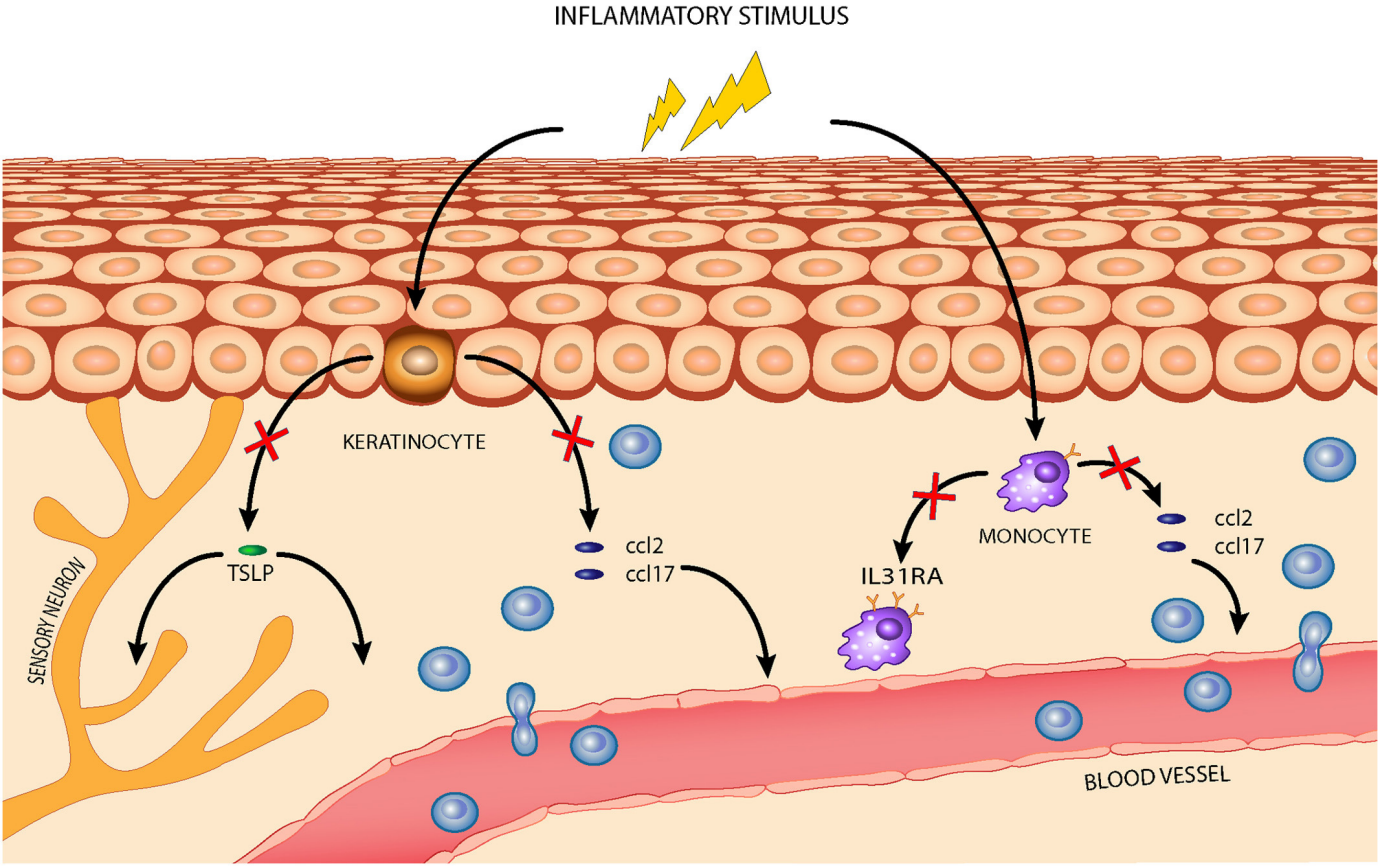
The effects of the nutraceutical mixture in a canine AD model: the nutraceutical mixture (red cross) inhibited the overexpression of homing chemokines *ccl2* and *ccl17* in stimulated CPEK cells, reducing leukocyte infiltration from blood vessels, and of *tslp*, reducing its activity in inflammatory cell recruitment and maturation. The nutraceuticals also decreased *ccl2* and *ccl17* expression in inflamed DH82 cells and downregulated *il31ra* expression, restraining Th2-mediated response amplification.

The first goal of our study was to identify a set of inflammation markers in an *in vitro* model of canine AD. Of relevance, the transcriptional regulation of many of these markers was modulated by the nutraceutical exposure. It is well-established that the regular use of dietary supplements, such as essential fatty acids (EFAs), polyphenols, probiotics, or vitamins, has benefits for atopic animals (28–32). Their mechanisms of action in canine AD are not completely elucidated but may involve binding of toll-like receptors and downregulation of the predominately Th2-mediated allergic response (3, 33, 34). The AD pharmacological treatments targeted the JAK/STAT pathway to prevent the downstream signaling of cytokines associated with the Th1/Th2 inflammatory response, an approach particularly supported by recent evidence which demonstrated the strong involvement of a Th1-mediated immune response (17, 35, 36). In this work, the tested mixture containing luteolin, piceatannol, and cannabidiol interfered with the expression of marker genes associated with both Th1- and Th2-mediated inflammation. In particular, it reverted *ccl2* and *ccl17* overexpression in both tested cell lines and also downregulated *tslp* expression in CPEK cells and *il31ra* expression in DH82 cells, as illustrated in Figure 7. The effects of the nutraceutical mixture were also evident with respect to *il4r* gene expression in CPEK cells and *tslp* gene expression in DH82 cells, but these effects were only time dependent. To the best of our knowledge, this is the first study in a canine *in vitro* model with a focus on the evaluation of epigenetic mechanisms at the basis of gene regulation evoked by the Th1/Th2 response and nutraceutical mixture treatment. CCL2 and CCL17 belong to a superfamily of secreted proteins involved in immunoregulatory and inflammatory processes driven by the Th1 and Th2 response, respectively (37). They are highly overexpressed in canine AD provoking leukocyte activation and infiltration at the inflammatory sites in several pathologies (7). They represent new target molecules for AD therapy, together with TSLP, IL-4, and IL-31, that could dampen Th2 responses regulating the immune cell proliferation and stimulation of sensory cutaneous neurons to transmit itch sensation (38, 39).

The data in literature related to *in vitro* and *in vivo* effects of polyphenols and cannabidiol on Th1 homing cytokines support our results (40, 41). In this regard, *ccl2* expression was inhibited in human keratinocytes and skin treated with polyphenols *in vitro* and *in vivo* (42, 43); in murine monocyte cell lines, *ccl2* blunting was observed after the luteolin treatment (44) and furthermore, piceatannol and cannabidiol are both capable of inhibiting *ccl2* expression in activated macrophages *in vitro* (45, 46). The ability of polyphenols and cannabidiol to modulate Th2 response mediators (47, 48), including CCL17 and TSLP in AD models (49, 50), is equally well-known. Nutraceutical effects on both Th1/Th2 inflammatory mediators are uncommon, especially in dogs, whereas this represents a target for treating inflammatory stress and allergies in humans (51–53). The use of different active ingredients contained in food allows regulation of the different inflammation pathways involved in the chronic complex diseases. The mechanisms through which polyphenols and cannabidiol exert their gene modulatory functions are various and include epigenetic modifications (54, 55). The results show that the tested nutraceutical mixture affected the cell lines differently, even inhibiting the same genes. The *ccl2* promoter was strongly methylated in monocytes, for both exposure times and in different sites, while the mixture did not exert any effect on *ccl2* promoter methylation in keratinocytes. In fact, our data agree with literature that *ccl2* gene promoter methylation is widely reported in human monocytes *in vitro* and *in vivo*, whereas no evidence can be found for the methylation of the *ccl2* promoter in keratinocytes (56, 57). This can be explained by taking into account that the transcriptional regulation of *ccl2*, in the context of non-resolving inflammation, appears to be more related to histone modification (58, 59). Methylation of the *ccl17* gene promoter affects both cell lines; an increase of *ccl17* expression *in vivo* can be reverted with food supplements administration in patients with AD (56, 57). *In vitro*, the transcriptional regulation of *ccl17* has been associated only with the transcriptional factor STAT6, which in turn plays a fundamental role in the activation of Th2 cells for ILs secretion (60). Of relevance, both animal and human studies suggest that functional nutrition can downregulate different proinflammatory transcriptional factors, including STAT6 (60). The association between *tslp* overexpression and AD onset has already been established *in vivo*, as well as its modulation through botanicals or probiotic supplementation (61, 62), Here, we demonstrated for the first time that the gene expression is ruled by epigenetic mechanisms; in fact, we observed an increase of the DNA methylation as a consequence of the nutraceutical mixture treatment. Likewise, literature reports less information on the transcriptional regulation of the *il31ra* gene despite its great relevance in AD, especially with regard to therapeutic applications; indeed, IL-31RA has such a pivotal role in the AD pathogenesis that an anti-IL-31RA monoclonal antibody was developed as a novel strategy for targeting pruritus associated with AD (63). Finally, it has been observed that Th2 cells *in situ* are exposed to considerable changes in chromatin architecture and gene expression (64–66), suggesting a strong influence of the environment on their activation, and therefore, the possibility of intervention with nutritional supplements.

The health impact of natural substances depends on their pharmacokinetic features, particularly pertinent when these are administered by the oral route. Most ingredients from botanicals demonstrate very poor absorption features, and are extensively metabolized by intestinal and hepatic enzymes and interact, with often unpredictable effects, with the gut microbiota (67). Thus, not knowing the precise oral bioavailability of the active ingredients of the tested mixture could be a limitation of the study here. After oral administration of *Chrysanthemum morifolium* extract to rats at 1.7 g/kg body weight, equivalent to 22.8 and 58.3 μmol/kg of luteolin and luteolin-7-O-glucoside, respectively, luteolin and its glycosides were quickly absorbed and luteolin, luteolin monoglucoside, and luteolin monoglucuronide were easily detected in the plasma with the highest levels of pure luteolin observed 1 h after oral administration, corresponding to 0.76 ± 0.27 μM (68). Although this result appears to be quite far from the dosage used here, the absorption rate of luteolin is known to be dose- and time- dependent with plasma concentrations, increasing several-fold after repeated administration or higher doses, especially if it is coadministered with other ingredients (69). Moreover, the levels of absorbed luteolin, detected in plasma in the free form, can reach the values used in our work (25 μM) by formulation with lipid carriers (70). With respect to piceatannol, the extent of its oral bioavailability characterizes this stilbene as also being an incompletely absorbed compound (71). In any case, in rats, the absolute oral bioavailability was as high as 50.7 ± 15% of the administered dose (72). Regarding cannabidiol, this lipophilic phytocannabinoid is poorly water soluble and subjected to extensive first-pass metabolism in the gastrointestinal tract, leading to a limited oral bioavailability of ~9% (73). However, natural substances, such as polyphenols, that can interfere with phase I and/or II enzymes and/or with enteric P-glycoprotein enhance its plasma levels in rats ~6-fold (73). Finally, among the three compounds tested in our investigation, cannabidiol is the only ingredient that has been studied in dogs, where its detected oral bioavailability ranged from 13 to 19% of the administered dose (74, 75). Therefore, despite our study being performed *in vitro*, these previously reported findings suggest that our results could be considered valid with respect to a nutraceutical approach to treating canine AD.

## Conclusion

Considering the role of the Th1/Th2 inflammatory response in the pathomechanism of AD, this canine model can be considered as an important development for the *in vitro* assessment of drugs and active ingredients. The tested nutraceutical mixture containing polyphenols and cannabinoids showed an ability to revert, through an epigenetic mechanism, the overexpression of canine AD key inflammatory genes, laying the groundwork for its application to sustained drug therapy or relapse prevention. The epigenetic modulation is involved in the induction, maintenance, heterogeneity, and recall response of memory T cells in Th1- and Th2-related immunity (76), enabling the management of chronic inflammation through nutraceuticals that modulate DNA methylation.

## Data Availability Statement

The raw data supporting the conclusions of this article will be made available by the authors, without undue reservation.

## Author Contributions

MM, CD'A, and MP: conceptualization and writing, reviewing, and editing. MM: methodology. MM, EDV, and BB: investigation. MM and MP: formal analysis and writing (original draft preparation). FDP: visualization. CR and CD'A: supervision and project administration. All authors have contributed to and have approved the final manuscript.

## Conflict of Interest

The authors declare that this study received funding from C.I.A.M. Srl. The funder has the following involvement with the study: the writing of this article and the decision to submit it for publication.
