# Supplementary material for: Polyphenols and Cannabidiol Modulate Transcriptional Regulation of Th1/Th2 Inflammatory Genes Related to Canine Atopic Dermatitis
Source: Front Vet Sci. 2021 Mar 5;8:606197. doi: 10.3389/fvets.2021.606197 (PMC7982812; doi:10.3389/fvets.2021.606197)
Supplement: Supplementary file 1 [file Data_Sheet_1.docx]

Supplementary Material

**Table S1.** DNA methylation (%) changes at modulated gene promoters after mixture treatment of CPEK cells.

| **CPEK** | | | | | | | | | |
| --- | --- | --- | --- | --- | --- | --- | --- | --- | --- |
| **Time** | | 4h | | | | 8h | | | |
| **Inflamed** | | no | | yes | | no | | yes | |
| **Mix** | | no | yes | no | yes | no | yes | no | yes |
| *ccl2* | site 1 | 9.18 ± 2.03 | 16.87 ± 9.20 | 8.33 ± 0.44 | 8.18 ± 0.24 | 8.01 ± 0.78 | 6.78 ± 2.53 | 5.15 ± 4.58 | 19.23 ± 9.23 |
|  | site 2 | 81.19 ± 2.53 | 85.60 ± 3.44 | 81.20 ± 2.00 | 82.68 ± 0.88 | 72.57 ± 13.83 | 81.25 ± 3.75 | 81.53 ± 0.60 | 59.00 ± 37.03 |
|  | site 3 | 73.55 ± 1.99 | 85.53 ± 14.42 | 73.81 ± 1.27 | 73.84 ± 1.08 | 73.55 ± 2.57 | 70.49 ± 5.80 | 65.18 ± 13.94 | 67.03 ± 17.83 |
|  | site 4 | 91.02 ± 0.53 | 86.34 ± 13.94 | 90.98 ± 0.83 | 91.76 ± 0.48 | 87.03 ± 7.46 | 89.35 ± 4.22 | 83.20 ± 15.53 | 81.54 ± 20.69 |
|  | site 5 | 90.27 ± 1.83 | 84.97 ± 15.39 | 91.23 ± 0.60 | 91.83 ± 1.70 | 85.22 ± 77.53 | 92.21 ± 2.07 | 86.71 ± 6.95 | 79.70 ± 16.44 |
|  | Average | 69.04 ± 0.96 | 67.29 ± 14.85 | 69.11 ± 0.65 | 69.66 ± 0.75 | 65.27 ± 6.83 | 68.02 ± 2.90 | 64.35 ± 11.55 | 61.30 ± 17.46 |
| *ccl17* | site 1 | 99.91 ± 0.15 | 99.11 ± 1.55 | 97.69 ± 2.33 | 99.33 ± 1.16 | 97.85 ± 1.00 | 98.84 ± 2.01 | 98.82 ± 1.11 | 100.00 ± 0.00 |
|  | site 2 | 93.17 ± 5.93 | 92.24 ± 7.24 | 88.55 ± 3.47 | 90.36 ± 2.33 | 86.14 ± 1.09 | 94.32 ± 5.09 | 92.02 ± 7.03 | **95.82 ± 3.66^e^** |
|  | site 3 | 69.74 ± 5.67 | 83.84 ± 3.49 | **81.44 ± 1.87^a^** | **75.22 ± 2.35^b,c^** | 80.94 ± 4.19 | 77.91 ± 8.50 | 78.09 ± 1.63 | 78.53 ± 7.99 |
|  | site 4 | 99.16 ± 1.46 | 99.16 ± 1.11 | 98.47 ± 1.80 | 100.00 ± 0.00 | 98.12 ± 1.80 | 99.75 ± 0.43 | 98.31 ± 2.92 | 92.56 ± 12.89 |
|  | site 5 | 86.42 ± 4.84 | 89.62 ± 3.66 | 88.78 ± 2.76 | 84.80 ± 7.61 | 87.29 ± 1.98 | 85.45 ± 5.83 | 88.41 ± 8.85 | 84.95 ± 4.45 |
|  | Average | 89.68 ± 0.73 | 92.79 ± 2.67 | 90.99 ± 0.93 | 89.94 ± 2.01 | 90.07 ± 0.31 | 91.25 ± 1.97 | 91.15 ± 4.05 | 90.37 ± 0.91 |
| *tslp* | site 1 | 96.12 ± 0.39 | 95.75 ± 1.53 | 95.06 ± 0.43 | **96.43 ± 0.44^c^** | 95.83 ± 1.59 | 97.66 ± 2.05 | 95.83 ± 1.79 | 96.85 ± 1.59 |
|  | site 2 | 95.03 ± 1.39 | 95.36 ± 1.79 | 93.97 ± 0.54 | 94.82 ± 0.58 | 93.33 ± 1.44 | 96.14 ± 2.00 | 96.05 ± 2.42 | 96.05 ± 2.42 |
|  | site 3 | 81.90 ± 1.95 | 82.46 ± 2.87 | 80.29 ± 0.94 | 81.21 ± 2.30 | 81.87 ± 1.19 | 79.93 ± 2.37 | 83.17 ± 2.78 | 83.17 ± 2.78 |
|  | site 4 | 87.10 ± 1.22 | 87.90 ± 3.96 | 85.38 ± 1.02 | 83.79 ± 4.17 | 86.23 ± 3.56 | 88.90 ± 5.00 | 87.72 ± 3.80 | 87.72 ± 3.80 |
|  | site 5 | 80.14 ± 4.65 | 80.62 ± 7.85 | 77.05 ± 1.10 | 71.25 ± 11.77 | 73.51 ± 2.90 | 77.37 ± 2.32 | 83.38 ± 9.04 | 83.38 ± 9.04 |
|  | site 6 | 93.94 ± 1.53 | 93.65 ± 0.74 | 92.01 ± 0.89 | 93.44 ± 0.35 | 92.59 ± 1.67 | 94.10 ± 0.63 | 93.66 ± 1.58 | 93.66 ± 1.58 |
|  | Average | 89.04 ± 1.88 | 89.33 ± 3.12 | 87.29 ± 0.31 | 86.82 ± 1.91 | 87.21 ± 1.00 | 89.02 ± 1.00 | 89.96 ± 1.83 | 90.14 ± 3.14 |

^a^ *p* < 0.05 vs no inflamed 4 h no mix 4 h; ^b^ *p* < 0.05 vs no inflamed 4 h yes mix 4 h; ^c^ *p* < 0.05 vs yes inflamed 4 h no mix 4 h; ^e^ *p* < 0.05 vs no inflamed 8 h no mix 8 h; ^f^ *p* < 0.05 vs yes inflamed 8 h no mix 8 h.

**Table S2.** DNA methylation (%) changes at modulated gene promoters after mixture treatment of DH82 cells.

| **DH82** | | | | | | | | | |
| --- | --- | --- | --- | --- | --- | --- | --- | --- | --- |
| **Time** | | 4h | | | | 8h | | | |
| **Inflamed** | | no | | yes | | no | | yes | |
| **Mix** | | no | yes | no | yes | no | yes | no | yes |
| *ccl2* | site 1 | 74.30 ± 16.90 | 86.19 ± 1.71 | 81.20 ± 6.39 | 87.95 ± 1.80 | 73.83 ± 3.24 | 83.91 ± 1.56 | 77.91 ± 5.97 | **77.43 ± 1.06^d^** |
|  | site 2 | 90.13 ± 4.19 | 91.73 ± 3.09 | 79.52 ± 6.16 | **91.46 ± 1.19^c^** | 71.47 ± 31.37 | 90.42 ± 2.07 | 87.31 ± 4.53 | 85.29 ± 5.13 |
|  | site 3 | 93.71 ± 3.19 | 92.44 ± 1.85 | 90.20 ± 4.96 | 94.93 ± 0.88 | 75.53 ± 28.30 | 92.56 ± 5.91 | 90.41 ± 5.32 | 93.13 ± 3.28 |
|  | site 4 | 93.79 ± 4.00 | 95.10 ± 0.69 | 92.49 ± 3.56 | **97.06 ± 0.85^b^** | 89.41 ± 14.96 | 96.73 ± 0.42 | 96.06 ± 0.38 | 96.34 ± 0.39 |
|  | site 5 | 88.88 ± 7.50 | 93.30 ± 1.44 | 93.51 ± 6.96 | 95.62 ± 1.52 | 89.62 ± 6.24 | 93.93 ± 0.27 | 92.15 ± 4.97 | 94.60 ± 1.71 |
|  | site 6 | 73.32 ± 4.23 | 72.30 ± 3.08 | 76.32 ± 2.95 | 75.23 ± 3.31 | 73.89 ± 1.82 | 77.48 ± 4.24 | 73.88 ± 4.11 | **84.39 ± 3.86^a,d,e,f^** |
|  | Average | 85.69 ± 5.19 | 88.51 ± 1.89 | 85.54 ± 3.69 | 90.65 ± 0.79 | 78.95 ± 16.98 | 87.51 ± 2.79 | 86.29 ± 3.30 | 88.53 ± 0.97 |
| *ccl17* | site 1 | 97.5 ± 3.54 | 98.93 ± 1.45 | 100.00 ± 0.00 | 99.38 ± 0.88 | 96.31 ± 5.55 | 99.35 ± 0.78 | 99.25 ± 1.30 | 100.00 ± 0.00 |
|  | site 2 | 82.92 ± 15.48 | 78.17 ± 1.01 | 93.46 ± 11.32 | 82.98 ± 15.70 | 77.29 ± 1.80 | 81.33 ± 0.01 | 81.25 ± 8.22 | 74.22 ± 2.79 |
|  | site 3 | 79.04 ± 2.98 | 83.23 ± 0.99 | 84.20 ± 2.40 | 81.70 ± 5.50 | 78.89 ± 7.28 | 83.87 ± 5.22 | **76.27 ± 1.33^b,c^** | **84.17 ± 0.97^a,f^** |
|  | site 4 | 99.73 ± 0.38 | 99.27 ± 1.26 | 99.95 ± 0.09 | 98.76 ± 2.15 | 99.63 ± 0.65 | 97.66 ± 1.62 | 100.00 ± 0.00 | 98.29 ± 2.20 |
|  | site 5 | 87.91 ± 3.67 | 88.24 ± 0.81 | 89.11 ± 1.75 | 89.12 ± 2.19 | 86.88 ± 4.53 | 91.32 ± 2.23 | 86.79 ± 1.81 | 89.60 ± 1.54 |
|  | Average | 89.42 ± 2.69 | 89.57 ± 0.26 | 93.34 ± 2.94 | 90.39 ± 5.18 | 87.80 ± 3.63 | 90.71 ± 2.88 | 88.71 ± 2.05 | 89.25 ± 2.38 |
| *il31ra* | site 1 | 49.52 ± 2.01 | 51.34 ± 2.50 | **44.88 ± 1.34^a^** | 49.25 ± 4.57 | 48.84 ± 1.91 | 50.16 ± 3.56 | 50.30 ± 3.27 | 48.46 ± 0.59 |
|  | site 2 | 47.50 ± 1.64 | 49.02 ± 1.69 | **43.07 ± 1.29^a^** | **47.21 ± 2.20^c^** | 47.04 ± 0.56 | 46.69 ± 0.54 | 47.46 ± 3.59 | 45.49 ± 1.92 |
|  | Average | 48.51 ± 1.11 | 50.18 ± 1.91 | 43.98 ± 0.86 | 48.23 ± 3.38 | 47.94 ± 1.12 | 48.42 ± 2.01 | 48.88 ± 3.38 | 46.98 ± 1.10 |

a p < 0.05 vs no inflamed 4 h no mix 4 h; b p < 0.05 vs no inflamed 4 h yes mix 4 h; c p < 0.05 vs yes inflamed 4 h no mix 4 h; d p < 0.05 vs yes inflamed 4 h yes mix 4 h; e p < 0.05 vs no inflamed 8 h no mix 8 h; f p < 0.05 vs yes inflamed 8 h no mix 8 h.
